# Optimal Roving Winding on Toroidal Parts of Composite Frames

**DOI:** 10.3390/polym15153227

**Published:** 2023-07-28

**Authors:** Jaroslav Mlýnek, Seyed Saeid Rahimian Koloor, Roman Knobloch

**Affiliations:** 1 Department of Mathematics, Faculty of Science, Humanities and Education, Technical University of Liberec, Studentská 2, 461 17 Liberec, Czech Republic; jaroslav.mlynek@tul.cz (J.M.); roman.knobloch@tul.cz (R.K.); 2 Composite Materials and Technical Mechanics, Institute of Aeronautical Engineering, Faculty of Mechanical Engineering, Universität der Bundeswehr München, Werner-Heisenberg-Weg 39, 85579 Neubiberg, Munich, Germany

**Keywords:** curved composite frame, roving winding, optimized winding procedure, winding angle, torus, straight helix, toroidal helix

## Abstract

Frames made of polymer composites are increasingly used in the aerospace, automotive, and agricultural industries. A frequently used technology in the production line of composite frames is winding rovings onto a non-load-bearing frame to form the structure using an industrial robot and a winding head, which is solidified through a subsequent heat-treatment pressure process. In this technology, the most difficult procedure is the winding of the curved parts of a composite frame. The primary concern is to ensure the proper winding angles, minimize the gaps and overlaps, and ensure the homogeneity of the wound layers. In practice, the curved frame parts very often geometrically form sections of a torus. In this work, the difficulty of achieving a uniform winding of toroidal parts is described and quantified. It is shown that attaining the required winding quality depends significantly on the geometrical parameters of the torus in question. A mathematical model with a detailed procedure describing how to determine the number of rovings of a given width on toroidal parts is presented. The results of this work are illustrated with practical examples of today’s industrial problems.

## 1. Introduction

The progress in the development of high-performance materials and structures has been a challenge that has required the development of novel materials with superior and selective mechanical properties and physical features to overcome the standard demand for quality and reliability at different scales [1,2,3]. The need to increase conventional material efficiency continues to focus materials development on the exploration of materials as composites [4,5]. In this regard, new polymer composites are developed to have special microstructures with unique features to replace conventional materials (e.g., steel, glass, wood) that are frequently used in the design of advanced structures. They are characterized above all by mechanical properties such as tensile, compressive, and torsional strength, lightness, long lifespan, and weather resistance. These exceptional mechanical properties make possible composite structures that can endure extreme loads and boundary conditions [4,6,7]. The important role of such composites in various applications that require the development of structures with complex geometries, such as profiles with open or closed cross-section forms, curved frames with rectangle to circular geometries, and antisymmetric planer shapes [8,9], forced designers to innovate many fabrication methods, such as the vacuum-infusion process, pultrusion process, and robot winding, enabling the possibility of such fabrications [10]. Examples for application of such composite structures are reinforcements for the fuselages, wings, and doors of aircraft, or the attachment part of windows to helicopter cockpits [11], or chassis reinforcements, car cabins, and door reinforcements in the automotive industry [12]. Composite frames (narrow curves, hollow structure) are utilized in many applications, such as orthopedic devices [13], the manufacture of sports equipment and bicycles [14], ship construction and fishery (hull reinforcements and masts) [5], internal parts of aircraft bodies [15], or to play the role of structural reinforcement [16]. As one of the important applications, such frame structures have been used in oil and petroleum industries as complex pipe or tank structures branching off different cross-sectional configurations of circular to elliptic shapes, utilized for transporting or storing oil and other petroleum liquid materials [17,18]. This is also due to the high potential of composite materials to bear severe loading under harsh environmental conditions. The composite frame structures with long wound fibers are normally fabricated using the filament-winding method by robot or machine to wind continuous strands of tow [19,20]. This winding process is highly adapted to arrange the fiber orientation in such a way that an ideal custom creation with lightweight structures is engineered to meet the desired strength characteristics as dictated by the application [21,22].

**Figure 1:**
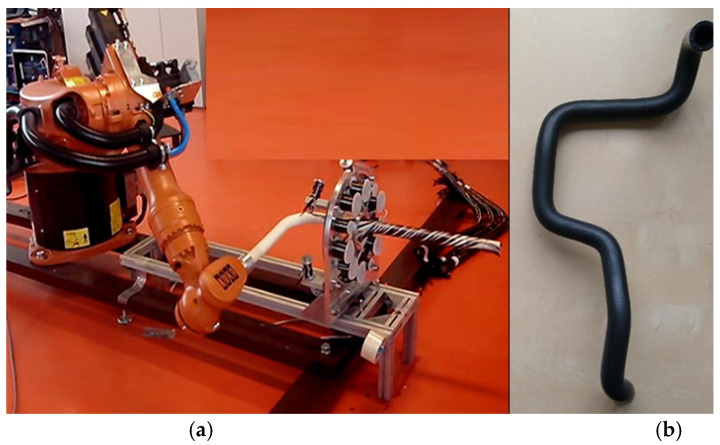
(**a**) The frame attached to the robot end effector passes through the winding head with a single rotating ring. One layer of winding is formed. (**b**) An example of a 3D frame with a complicated shape.

**Figure 2:**
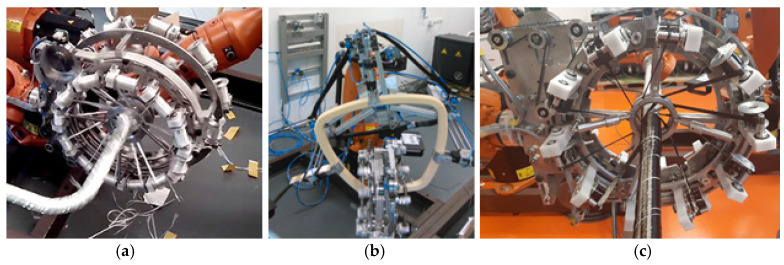
(**a**) Simultaneous winding of three layers of glass rovings on the open frame. (**b**) Fixing the closed frame to the robot end effector. (**c**) Rotating ring of winding head with coils with wound rovings.

In robot filament winding, the winding of rovings on a non-load-bearing frame is performed by a winding head and an industrial robot (see Figure 1a). The frame is generally 3D; it can also have a geometrically complicated shape (see Figure 1b). The frame is attached to the end of the robot’s working arm (robot end effector; see Figure 1a). The winding head contains usually three rotating rings (see Figure 2a). Several coils with rovings are placed evenly around the circumference of each ring (see Figure 2c). 

Based on the determination of a suitable robot trajectory, the frame passes through the winding head at a constant speed. Each of the three rings performs the winding of a layer of rovings; based on the determination of the necessary angular speed of each ring (control provided by the robot’s external axis), each layer is wound at the specified angle. Three layers of windings at different angles are thus created in one pass of the frame through the winding head. A detailed description of the calculation of the optimized robot trajectory is given in [23].

**Note 1.**

Roving is a fiber system that enables single filaments to be arranged in one parallel collection without twists. Fiber rovings (from carbon, glass, basalt, or aramid fibers) are used to produce 3D composite reinforcement.

Both open and closed frames can be wound using this winding procedure (see Figure 2a,b).

The quality of the composite frame significantly depends on maintaining the required winding angles, and ensuring the homogeneity of the windings (i.e., roving windings without overlaps and gaps). This article focuses on the quality of winding of the composite frame from a geometric perspective. At the same time, the quality of the composite also depends on the material properties of the rovings (e.g., rovings from carbon, glass, aramid, and rovings from recycled materials). However, studying the properties and quality of the fibers used in rovings is not included in the article. 

Ideal roving winding can be formed on a frame with a circular cross-section if it forms a straight segment. In this case, a smooth, high-quality winding of the roving onto the frame can be realized. However, winding the curved parts of the frame is more difficult when high-quality winding is required. Simultaneous testing and ensuring the collision-free passage of the frame through the winding head is essential for 3D frames [23,24]. 

A constant speed of the frame through the winding head is assumed during the winding process. The winding angle is regulated by changing the angular speed of the winding rotated ring of the head. This issue is discussed in detail in [25]. It is also possible to calculate the distance of the roving winding on the frame from the rotating ring (this distance depends on the specified winding angle, the radius of the ring, and the radius of the wound frame; for detail see [25]). This enables smooth and continuous change from a given winding angle to another. The optimization of the number and width of the rovings used for specific winding is discussed in [26]. Based on this optimization, the formation of gaps and overlaps is minimized during the winding process. 

As already mentioned, winding the curved parts of the frames is the most difficult process of winding technology. The curved sections of the frame often form parts of a *torus* (see next figures). Based on the literature review and to the best of the authors’ knowledge, such a study has not been undertaken before. Therefore, this study focuses on the procedures for the optimal winding of the curved sections of frames shaped like parts of a torus. It is highlighted that achieving acceptable winding quality depends on the torus geometry. A mathematical model of the winding procedure and a detailed analysis of the possibility of achieving an acceptable and optimized winding of the frame with toroidal parts is described in the next sections. In addition, practical examples of the application of the various torus geometries are provided.

## 2. Materials and Methods

**Figure 3:**
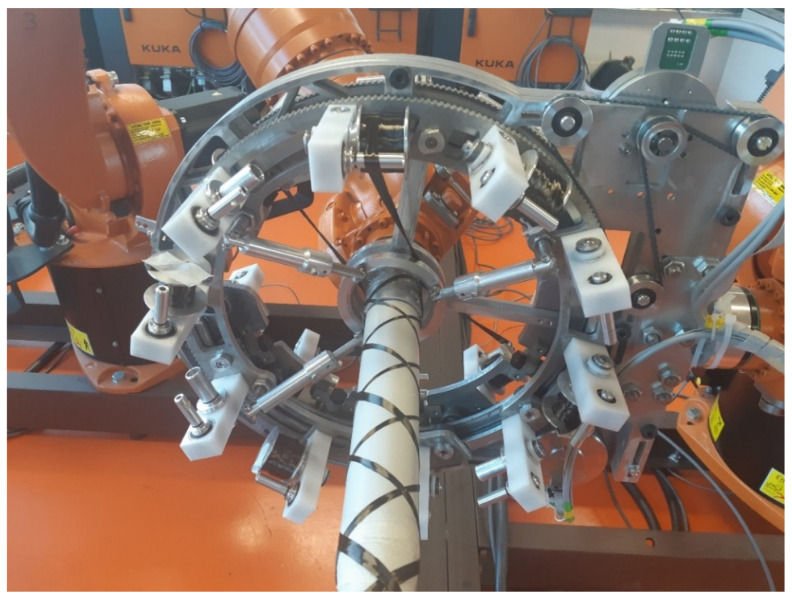
The first rotating ring of the winding head winds one roving at an angle of 45° and the following second rotating ring winds the roving at an angle of −45°.

Winding the roving onto a straight frame of circular cross-section using a winding head creates a helix on the surface of the frame (see Figure 3). A standard helix wound on a straight frame is called a *straight helix*.

**Figure 4:**
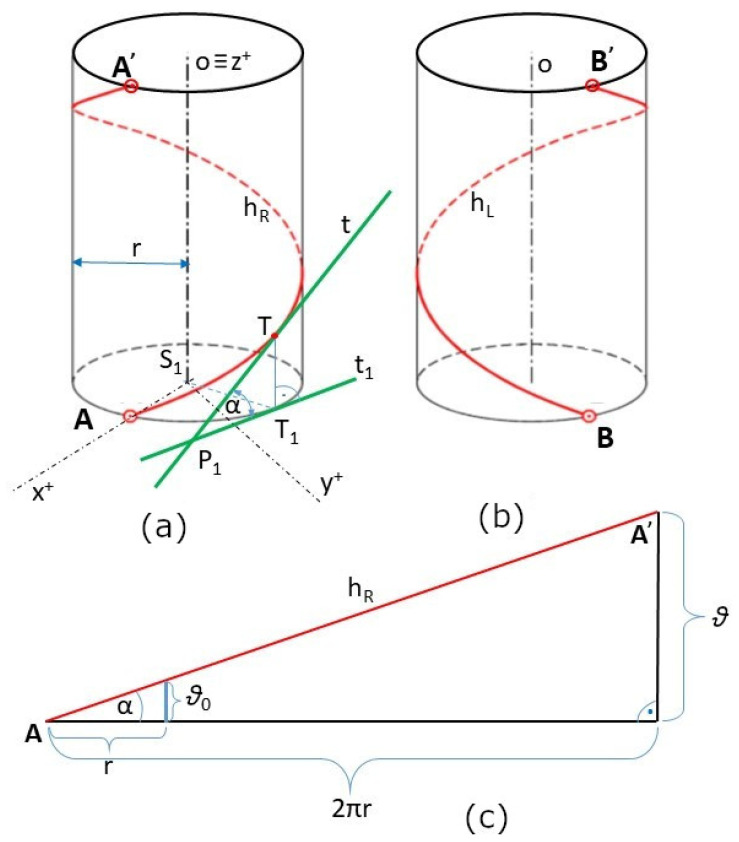
(**a**) One turn of a right-hand straight helix $h_{R}$. (**b**) One turn of a left-hand straight helix $h_{L}$. (**c**) Characteristics triangle of a straight helix.

If the wound roving forms a right-handed straight helix on the surface of the frame, it is said to have a positive winding angle (see Figure 4a). If a left-hand straight helix is formed, this is interpreted as a negative winding angle (see Figure 4b). One turn of the straight helix is shown in both cases Figure 4a,b. The following sections focus only on the winding in a positive direction and the creation of a right-handed straight helix. Winding at a negative angle is completely analogous.

One turn of right-handed straight helix $h_{R}$ (initial point A and endpoint A’) is shown in Figure 4a. This straight helix is defined by its axis *o* (longitudinal axis of the frame), radius *r* (radius of the frame), and pitch ϑ (height of one helix turn measured parallel to axis o of the helix), which is the Euclidean distance between points A and A’ in Figure 4a; for detail see [27]. A characteristic triangle (see Figure 4c) defines the straight helix angle α, where

(1) $$tg\alpha =\frac{\vartheta }{2\pi r}=\frac{\vartheta_{0}}{r}\;.$$

Parameter ϑ is a pitch of straight helix per 2π, and parameter $\vartheta_{0}$ is a pitch per one radian. Angle α is defined as an angle between tangent *t* to $h_{R}$ at point T of the straight helix and its orthogonal projection $t_{1}\;$into the ground plane (see Figure 4a). In the following, we will call the angle α defined by Relation (1); the winding angle. It is true that $\alpha \in \left(0,\;\right.\;\left.\pi /2\right\rangle$. In case of α=π/2, the roving is laid parallel to the axis o and longitudinal to the frame surface.

Specialists in the field of composite materials often call the β angle; the winding angle, as defined by the relationship

(2) $$\beta =\frac{\pi }{2}-\alpha .$$

In this article, the winding angle will mean the angle α defined by Relation (1).

### 2.1. Torus-Shaped Part of the Frame

**Figure 5:**
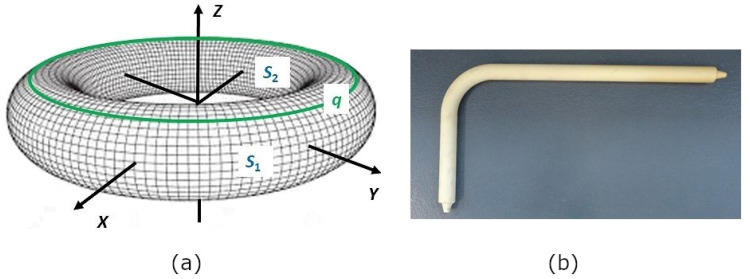
(**a**) Model of the torus. (**b**) Non-bearing polyurethane frame for winding rovings with a middle section forming part of the torus.

As already mentioned in the introduction of the article, winding the curved sections of frames belongs to the most difficult part of the winding technology when using a winding head and an industrial robot. These frame sections often form parts of a torus (see Figure 5). 

Instead of describing the winding of the curved part of the frame in the shape of a torus, for simplicity, we provide a description of the procedure for the case of winding rovings on the whole torus.

**Figure 6:**
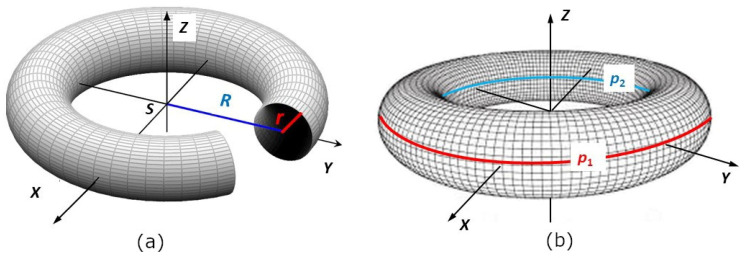
(**a**) An example of a torus. (**b**) Torus with outer circumferential circle $p_{1}$ and inner circumferential circle $p_{2}$.

From a geometric point of view, a torus is a 3D body created by rotating a circle of radius *r* around a line lying in the plane of this circle and not intersecting this circle (see Figure 6a). The center of the circle of radius *r* is placed on the *y*-axis and its distance from origin *S* of the coordinate system is *R*, where 0<r<R. Rotation of this circle around axis z creates a torus (see Figure 6a). The value of R is called the *major radius* and r the *minor radius* of the torus.

**Figure 7:**
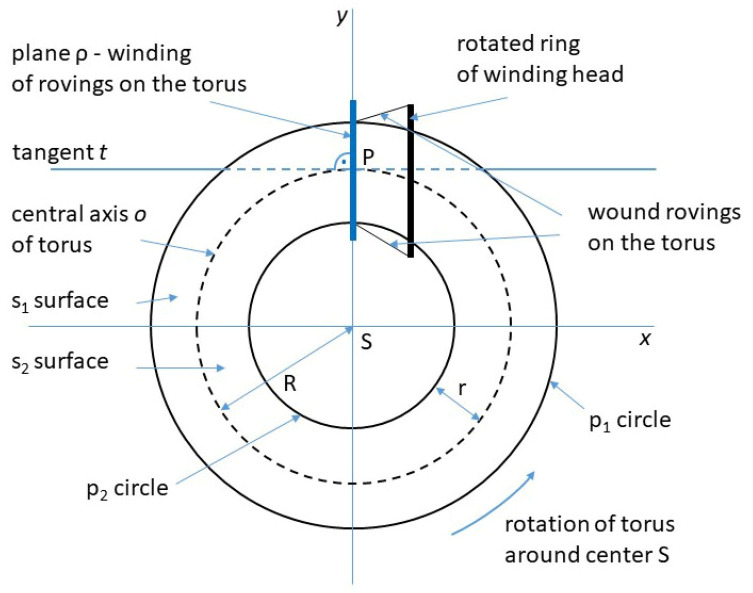
Schematic ground plan of the torus, its parameters, and rotated ring of winding head.

Similar to winding a roving on a straight frame at the α angle, it is necessary that the tangent *t* at point P of the intersection of frame axis o and plane ρ of winding of the roving on the frame is orthogonal to plane ρ (see Figure 7).

However, the roving is not wound at the specified constant α angle. The winding angle of the roving changes continuously during one turn (it is described in more detail in Section 2.3 and Section 2.4; see also [28]).

### 2.2. Level of Difficulty of Roving Winding

In this paragraph, the focus is on determining the difficulty of winding the roving onto a torus.

The *aspect ratio* a of the torus is defined as

(3) a=r/R. 

The aspect ratio a significantly affects the difficulty of winding the torus. The smaller the value of a, the easier the torus can be wound. In the case of a→0, the torus transforms into a straight cylinder.

**Figure 8:**
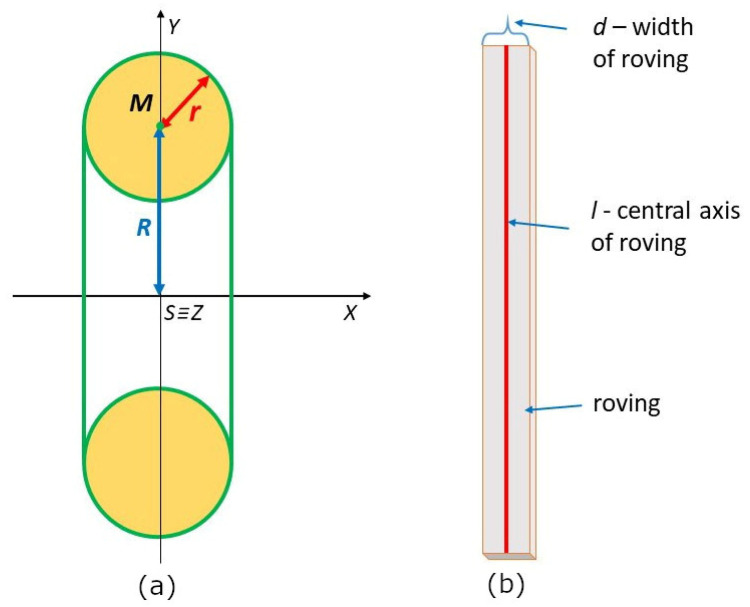
(**a**) Torus centered at the origin S, xy-plane cut, rotation of circle k  ≡ (*M*, *r*) around the *x*-axis. (**b**) Roving of width *d* with the central axis *l*.

A vertical cut through the circle q (see Figure 5b) divides the surface $s_{total}$ of the whole torus into outer part $s_{1}$ and inner part $s_{2}$ (see Figure 5a and Figure 7). The winding difficulty is caused by the different surface sizes of part $s_{1}$ and part $s_{2}$ of the torus. Integral calculus is used to determine $s_{1}$ and $s_{2}$ (see Figure 8a). Radius R+r of circle $p_{1}$ (see Figure 6a and Figure 7) is called the *outer radius* of the torus and radius R−*r* of circle $p_{2}$ the *inner radius* of the torus. 

Surface $s_{total}$ of the whole torus is composed of partial surfaces $s_{1}$ and $s_{2}$, i.e., $s_{total}=s_{1}+s_{2}$, and (see [29], p. 26)

(4) $$s_{total}=4\pi^{2}rR\;.\;$$

**Figure 9:**
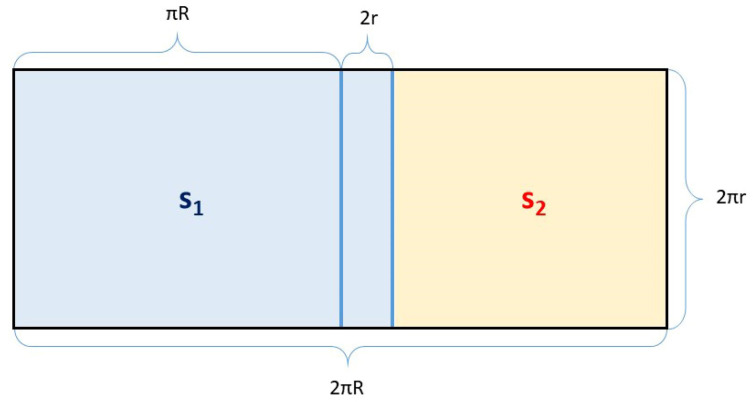
Graphical representation of the ratio of the size of partial surfaces $s_{1}$ and $s_{2}$, and $s_{total}=s_{1}+s_{2}$.

Surface $s_{total}$ of the whole torus is thus equal to the contents of a rectangle with the lengths of the sides 2*πR* and 2*πr* (see Figure 9).

In addition, the focus is concentrated on the calculation of values $s_{1}$ and $s_{2}$. Surface $s_{1}$ is created by rotating the curve $f\left(x\right)=R+\sqrt{r^{2}-x^{2}}$ around the x-axis, where x∈<−r, r> (see [30], p. 107; Figure 8a). Therefore, the size of surface $s_{1}$ can be calculated by the following procedure:

$$\begin{array}{l} s_{1}=2\pi \;\int_{-r}^{r}f\left(x\right)\sqrt{1+{\left(f^{\prime }\left(x\right)\right)}^{2}}dx=2\pi \int_{-r}^{r}(R+\sqrt{r^{2}-x^{2}})\cdot \sqrt{1+{\left(\frac{-x}{\sqrt{r^{2}-x^{2}}}\right)}^{2}}\;dx=\\ =2\pi \int_{-r}^{r}(R+\sqrt{r^{2}-x^{2}})\sqrt{1+\frac{x^{2}}{r^{2}-x^{2}}}dx=2\pi \int_{-r}^{r}(R+\sqrt{r^{2}-x^{2}})\cdot \sqrt{\frac{r^{2}-x^{2}+x^{2}}{r^{2}-x^{2}}}dx=\\ =2\pi \int_{-r}^{r}(R+\sqrt{r^{2}-x^{2}})\cdot \frac{r}{\sqrt{r^{2}-x^{2}}}dx=2\pi Rr\int_{-r}^{r}\frac{1}{\sqrt{r^{2}-x^{2}}}dx+2\pi r\int_{-r}^{r}1dx=\\ =2\pi {\left[Rr\;arcsin\frac{x}{r}+rx\right]}_{-r}^{r}=2\pi \left[Rr\;arcsin1+r^{2}-\left(Rr\arcsin\left(-1\right)-r^{2}\right)\right]=\\ =2\pi \left[Rr\;\frac{\pi }{2}+r^{2}-\left(Rr\left(-\frac{\pi }{2}\right)-r^{2}\right)\right]=2\pi^{2}Rr+4\pi r^{2}. \end{array}$$

In the previous derivation, the relation $\int \frac{dx}{\sqrt{r^{2}-x^{2}}}=\arcsin\frac{x\;}{r}$ (see [30], p. 150) is used. This relationship also follows from the derivative of composite function $arcsin\frac{x}{r}$:

$${(\arcsin\frac{x}{r})}^{\prime }=\frac{1}{\sqrt{1-\frac{x^{2}}{r^{2}}}}\;\cdot \;\frac{1}{r}=\frac{1}{\sqrt{r^{2}}\sqrt{1-\frac{x^{2}}{r^{2}}}}=\frac{1}{\sqrt{r^{2}-x^{2}}}.$$

The size of the surface $s_{1}$ is therefore given by the relation

(5) $$s_{1}=2\pi^{2}Rr+4\pi r^{2}.$$

Since the following holds: $s_{2}=s_{total}-s_{1}$, and from the Relations (4) and (5), this implies

(6) $$s_{2}=2\pi^{2}Rr-4\pi r^{2}.$$

Thus, the value for the ratio $s_{2}/s_{1}$ and the use of Relations (5) and (6) is equal to

(7) $$\frac{s_{2}}{s_{1}}=\frac{2\pi^{2}Rr-4\pi r^{2}}{2\pi^{2}Rr+4\pi r^{2}}=1-\frac{8\pi r^{2}}{2\pi^{2}Rr+4\pi r^{2}}=1-\frac{4r}{\pi R+2r}<1.\;$$

The more the ratio $s_{2}/s_{1}\;$in Relation (7) approaches the value 1, the more acceptable the conditions for the winding of rovings are. It follows from Relation (7) that the larger the value of *R* with respect to *r*, the better the conditions for roving winding. The sizes of the areas corresponding to $s_{total}$, $s_{1}$, and $s_{2}$ are shown graphically in Figure 9.

Figure 9 shows that the geometrical conditions for roving winding are better the smaller the blue marked area of size 2r·2πr.

As stated in [26], circumference $o(p_{1})$ of the *outer circumferential circle* $p_{1}$ (see Figure 6b and Figure 7) is equal to $o(p_{1})=2\pi (R+r)$ and circumference $o(p_{2})$ of inner circumferential circle $p_{2}$ is equal to $o(p_{2})=2\pi (R-r)$, while it is valid R>r (see Figure 6). This then implies

(8) $$\frac{{o(p}_{2})}{{o(p}_{1})}=\frac{2\pi (R-r)}{2\pi (R+r)}=\frac{R-r}{R+r}=\frac{R+r}{R+r}-\frac{2r}{R+r}=1-\frac{2r}{R+r}<1.$$

It follows from Relation (8) that winding of the torus is easier the closer the $o(p_{2})/o(p_{1})$ ratio is to 1, i.e., the smaller the positive value of 2r/(R+r). 

Relations (7) and (8) characterize the difficulty of winding rovings on the curved part of the frame.

Thus, it follows from Relations (7) and (8) that the smaller the value of aspect ratio a defined by Relation (3) (i.e., R≫r), the more homogeneous the winding that can be achieved. 

### 2.3. Mathematical Description of Roving Winding on the Torus

Our attention in this paragraph is focused on the procedure of winding rovings onto the surface of a torus. In the next mathematical model of the roving winding on the surface of the torus, only the central axis *l* (see Figure 8b) of the roving will be considered. 

The torus can be parametrically defined in a 3D right-handed Euclidean space in the form (see [31], p. 65)

(9) $$\begin{array}{l} x\left(\theta ,\;\varphi \right)=\left(R+r\;cos\theta \right)\;cos\varphi ,\\ y\left(\theta ,\;\varphi \right)=\left(R+r\;cos\theta \right)\;sin\varphi ,\\ z\left(\theta ,\;\varphi \right)=r\sin\theta . \end{array}$$

Recall that major radius *R* denotes the radius of the central axis *o* of the torus (see Figure 7) and the minor radius *r* the radius of the tube (see Figure 6a, Figure 7 and Figure 8a). Parameters θ and φ are the angles that make the whole torus, θ, φ ∈<(0, 2π). Angle θ represents rotation around the tube, whereas φ represents rotation around the torus’s central axis o (see Figure 7).

**Figure 10:**
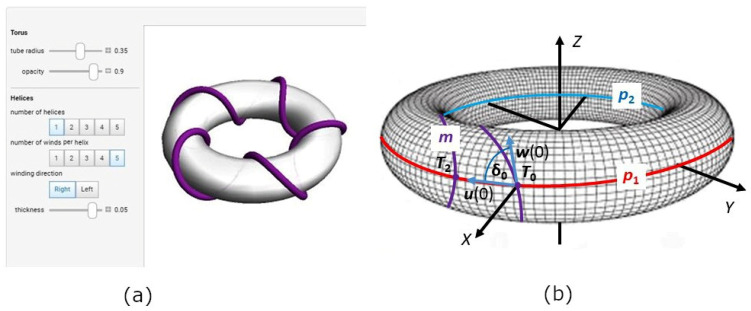
(**a**) Graph of right-handed toroidal helix for specified parameters R=100, r=33, ω=5 (number of winds per helix). (**b**) δ angle clamped by vectors u(0) and w(0) at point $T_{0}$ of toroidal helix δ. (Figure 10a and Figure 12 are generated by “Toroidal Helices—Wolfram Demonstrations Project” graphics application freely available from https://www.google.com/search?q=toroidal-helix&oq=toroidal-helix&aqs=chrome..69i57j0i13i30.10920j0j15&sourceid=chrome&ie=UTF-8#imgrc=HAw5MhPvHq4pfM, accessed on 11 June 2023).

The parametric expression of a right-handed helix wound on a torus can be expressed in the following form [28]

(10) $$\begin{array}{l} x\left(t\right)=\left(R+r\cos\left(\omega t\right)\right)\cos t,\\ y\left(t\right)=-\left(R+r\cos\left(\omega t\right)\right)\sin t,\\ z\left(t\right)=r\;sin(\omega t) \end{array}$$

for t∈R, ω is a real positive constant; parameters R and *r* have the same meaning as in Relation (9). This winding defined by Relation (10) describes the helix wrapped around the torus and is called the right-handed *toroidal helix* (see Figure 10a). 

When ω is a natural number, the toroidal helix creates a closed loop and ω defines the number of times the toroidal helix coils around the torus (in more detail see [28]). The circumference O(o) of the central axis o of the torus (see Figure 7) is equal to $O\left(o\right)=2\pi R$. Following this, toroidal pitch H (specifies the length of repetition along the center axis o) and corresponding reduced toroidal pitch $H_{0}$ (pitch per one radian) are defined by the relations

(11) $$H=\frac{2\pi R}{\omega },\;H_{0}=\frac{H}{2\pi }=\frac{R}{\omega }.$$

The central axis o of the torus passes at the same speed through the winding head as in the case of a straight frame and this and Relation (1) imply that for toroidal pitch,

(12) H=ϑ=2πr tgα0.

Recall that α indicates the winding angle on the straight part of the frame.

### 2.4. Determination of Winding Angle on Torus

When the roving is wound onto a straight frame, a straight helix is formed with the same winding angle at all points of the resulting helix. When winding the toroidal helix, however, the winding angle continuously changes. This paragraph focuses on a more detailed description of the winding angle in the case of the toroidal helix.

A torus with major radius R and minor radius r in Figure 6a and Figure 7 has its center *S* placed at the origin in the 3D right-handed Euclidean coordinate system. Circles $p_{1}$, $p_{2}$, and central axis o lie in the plane defined by the *x* and *y* axes. The points of the wound toroidal helix defined by Relation (10) and lying on the circles $p_{1}$ or $p_{2}$ can be determined by the following procedure. The z-coordinate of these points is zero. Therefore, it follows from Relation (10)

$$z\left(t\right)=r\;\sin\left(\omega t\right)=0.$$

The relationship is valid when $\sin\left(\omega t\right)=0$, which implies ωt=k.π, where k is an arbitrary integer number. From here it follows

(13) $$t=\frac{k.\pi }{\omega }.$$

Applying Relation (13) successively for k=0, 1, 2, 3, it follows $t_{0}=0,t_{1}=\pi /\omega ,t_{2}=2\pi /\omega$, and $t_{3}=3\pi /\omega$. Points of toroidal helix $T_{0}=\left[x\left(t_{0}\right),y\left(t_{0}\right),z(t_{0})\right]$, $T_{2}=\left[x\left(t_{2}\right),y\left(t_{2}\right),z(t_{2})\right]$ lie on the outer circle $p_{1}$ of torus and points $T_{1}\left[x\left(t_{1}\right),y\left(t_{1}\right),z(t_{1})\right]$, $T_{3}\left[x\left(t_{3}\right),y\left(t_{3}\right),z(t_{3})\right]$ lie on the inner circle $p_{2}$ of the torus. The components of these points can be expressed using the relationship (10):

(14) $$\begin{array}{l} T_{0}=\left[x\left(t_{0}\right),y\left(t_{0}\right),z(t_{0})\right]=\left[R+r,\;0,\;0\right]\;,\\ \;\;T_{1}\left[x\left(t_{1}\right),y\left(t_{1}\right),z(t_{1})\right]=\left[\left(R-r\right)cos\frac{\pi }{\omega },-\left(R-r\right)sin\frac{\pi }{\omega },\;0\right],\\ T_{2}=\left[x\left(t_{2}\right),y\left(t_{2}\right),z(t_{2})\right]=\left[\left(R+r\right)cos\frac{2\pi }{\omega },-\left(R+r\right)sin\frac{2\pi }{\omega },\;0\right],\\ \;\;T_{3}\left[x\left(t_{3}\right),y\left(t_{3}\right),z(t_{3})\right]=\left[\left(R-r\right)cos\frac{3\pi }{\omega },-\left(R-r\right)sin\frac{3\pi }{\omega },\;0\right]. \end{array}$$

Attention is focused on determining the winding angle on the outer circumference of the torus (circle $p_{1}$) and on the inner circumference of the torus (circle $p_{2}$). The tangent vector w(t) at any point of the toroidal helix can be obtained by the following procedure. From Relation (10) it follows

(15) $$\begin{array}{l} \frac{\partial x}{\partial t}=r\cdot \left(-\sin\left(\omega t\right)\right)\cdot \omega \cdot \cos t+(R+r\cdot \cos(\omega t))\cdot (-\sin t)=\\ \;\;\;\;\;\;\;\;\;=-r\omega \sin\left(\omega t\right)\cdot \;\cos t-\left(R+rcos\;\left(\omega t\right)\right)\cdot \sin t,\\ \;\;\;\;\;\;\frac{\partial y}{\partial t}=-[r\cdot (-\sin\left(\omega t\right)\cdot \omega \cdot \sin t+(R+r\cdot \cos(\omega t))\cdot \cos t]=\\ \;\;\;\;\;\;\;\;\;=+r\omega (\sin(\omega t))\cdot \sin t-(R+r\cdot \cos(\omega t))\cdot \cos\;t,\\ \;\;\;\;\;\;\;\;\;\;\;\;\;\;\;\;\;\frac{\partial z}{\partial t}=r\cdot \cos\left(\omega t\right)\cdot \omega =r\omega \cdot \cos\left(\omega t\right). \end{array}$$

The tangential direction vector w(t) at the point $\left[x\left(t\right),y\left(t\right),z(t)\right]$ has the expression

(16) $$w(t)=\left(\frac{\partial x}{\partial t},\;\frac{\partial y}{\partial t},\;\frac{\partial z}{\partial t}\right)\;,$$

where $\frac{\partial x}{\partial t},\;\frac{\partial y}{\partial t}$, and $\frac{\partial z}{\partial t}$ are defined by Relation (15).

Point $T_{0}\;$lies on the circle $p_{1}$ and has coordinates $T_{0}=\left[R+r,\;0,\;0\right]\;$ according to Relation (14).

Tangent vector $u(t_{0})$ to the circle $p_{1}$ at point $T_{0}$ lying in the plane of the *x* and *y* axes (ground plane) can then be expressed in the form $u(t_{0})=\left(0,\;R+r,\;0\right)$; see Figure 10b.

Recall that in Euclidean space $E_{3}$, the length of the vector $\vec{u}$ is defined by the relation $\left\|u\right\|=\sqrt{x_{u}^{2}+y_{u}^{2}+z_{u}^{2}}$. The scalar product u.v of vectors u and v is defined by $u.v=x_{u}\cdot x_{v}+y_{u}\cdot y_{v}+z_{u}\cdot z_{v}$. The tangent vector $w(t_{0})$ to the toroidal helix at point $T_{0}$ is according to Relations (15) and (16) of the form $w\left(t_{0}\right)=w\left(0\right)=\left(0,-(R+r),\;r\omega \right).$ The angle $\delta_{0}$ enclosed by the vectors u(0) and w(0) can be determined using the relation (see [32], p. 113)

$$\cos\delta_{0}=\frac{\vec{u}(0)\times \vec{w}(0)}{\left\|\vec{u}(0)\right\|\times \left\|\vec{w}(0)\right\|}=\frac{\left(0,-(R+r),\;0\right)\cdot \;(0,-(R+r),\;r\omega )}{\left(R+r\right)\cdot \sqrt{{\left(R+r\right)}^{2}+r^{2}\omega^{2}}}=\frac{R+r}{\sqrt{{\left(R+r\right)}^{2}+r^{2}\omega^{2}}},$$

thus

(17) $$\delta_{0}=\arccos\left(\frac{R+r}{(\sqrt{{\left(R+r\right)}^{2}+r^{2}\omega^{2}}}\right).$$

Similarly, tangent vector $w\left(t_{1}\right)\;$to the toroidal helix at point $T_{1}$ is $w\left(t_{1}\right)=w\left(\frac{\pi }{\omega }\right)=\left(-\left(R-r\right)sin\frac{\pi }{\omega },-\left(R-r\right)cos\frac{\pi }{\omega },-r\omega \right).$ Tangent vector ${u(t}_{1})$ to the circle $p_{2}$ at point $T_{1}$ lying in the plane of the *x*, *y* axes (ground plane) can be expressed in the form ${u(t}_{1})=\left(R-r\right)\cdot \left(-sin\frac{\pi }{\omega },-cos\frac{\pi }{\omega },\;0\right).$ Thus, it is true for the angle between vectors $u_{1}$ and $w\left(t_{1}\right)$

$$\cos\delta_{1}=\frac{u(t_{1})\times w(t_{1})}{\left\|{u(}_{t_{1}})\right\|\times \left\|w(t_{1})\right\|}=\frac{\left(R-r\right)\cdot \left(-sin\frac{\pi }{\omega }\;,-cos\frac{\pi }{\omega },\;0\right)\cdot \;\left(-\left(R-r\right)sin\frac{\pi }{\omega },-\left(R-r\right)cos\frac{\pi }{\omega },-r\omega \right)}{\sqrt{{\left(R-r\right)}^{2}\left(sin\frac{\pi }{\omega }+cos\frac{\pi }{\omega }\right)}\;\cdot \;\sqrt{{\left(R-r\right)}^{2}{(sin}^{2}\frac{\pi }{\omega }+{cos}^{2}\frac{\pi }{\omega })+r^{2}\omega^{2}}}=\;\frac{{\left(R-r\right)}^{2}}{(R-r)\sqrt{{\left(R-r\right)}^{2}+r^{2}\omega^{2}}}=\frac{R-r}{\sqrt{{\left(R-r\right)}^{2}+r^{2}\omega^{2}}}.$$

Thus it is that

(18) $$\delta_{1}=\arccos\left(\frac{R-r}{(\sqrt{{\left(R-r\right)}^{2}+r^{2}\omega^{2}}}\right).$$

**Note 2.**

Let R, r, ω be real numbers and R>r. Thus,

(19) $$\frac{R+r}{\sqrt{{\left(R+r\right)}^{2}+r^{2}\omega^{2}}}>\frac{R-r}{\sqrt{{\left(R-r\right)}^{2}+r^{2}\omega^{2}}}.$$

**Proof.**  Assume the validity of Relation (19). After the removal of fractions, partial adjustments of the inequality are gradually made

$$\frac{R+r}{(\sqrt{{\left(R+r\right)}^{2}+r^{2}\omega^{2}}}>\frac{R-r}{\sqrt{{\left(R-r\right)}^{2}+r^{2}\omega^{2}}}$$

$$\left(R+r\right)\cdot \;\sqrt{{\left(R-r\right)}^{2}+r^{2}\omega^{2}}>\left(R-r\right)\cdot \;\sqrt{{\left(R+r\right)}^{2}+r^{2}\omega^{2}}$$

$${\left(R+r\right)}^{2}\cdot \;({\left(R-r\right)}^{2}+r^{2}\omega^{2})>{\left(R-r\right)}^{2}\cdot \;({\left(R+r\right)}^{2}+r^{2}\omega^{2})$$

$${\left(R+r\right)}^{2}\cdot {\left(R-r\right)}^{2}+{\left(R+r\right)}^{2}r^{2}\omega^{2}>{\left(R-r\right)}^{2}\cdot \;{\left(R+r\right)}^{2}+{\left(R-r\right)}^{2}r^{2}\omega^{2}$$

$${\left(R+r\right)}^{2}r^{2}\omega^{2}>{\left(R-r\right)}^{2}r^{2}\omega^{2}$$

$${\left(R+r\right)}^{2}>{\left(R-r\right)}^{2}$$

The last inequality holds for arbitrary real numbers R, r for R>r. From Relation (19) and the fact the arccos function is decreasing in the interval $\langle 0,\;1\rangle$, it follows that $\delta_{1}>\delta_{0}$. In accordance with Relation (2), it follows that ${\tilde{\alpha }}_{0}=\frac{\pi }{2}-\delta_{0}$, ${\tilde{\alpha }}_{1}=\frac{\pi }{2}-\delta_{1}$ and it is true ${\tilde{\alpha }}_{0}>{\tilde{\alpha }}_{1}$. The transition from the point $T_{0}$ to point $T_{2}$ on the circle $p_{1}$ is made at one turn of the filament on the outer circumference of the torus. Analogously, transition from the point $T_{1}$ to $T_{3}$ on the circle $p_{2}$ is made also in one turn filament on internal circumference of the torus. This means that on the outer circumference of the torus, the filament is wound at an angle

(20) $${\tilde{\alpha }}_{ext}=\frac{\pi }{2}-\delta_{0}$$

and in the internal circumference of the torus, the filament is wound at an angle

(21) $${\tilde{\alpha }}_{int}=\frac{\pi }{2}-\delta_{1}.$$

At the same time, ${\tilde{\alpha }}_{int}<$ ${\tilde{\alpha }}_{ext}$ and the filament winding angle $\tilde{\alpha }$ varies continuously over the interval $\langle {\tilde{\alpha }}_{int},\;{\tilde{\alpha }}_{ext}\rangle$. When winding the filament on a straight frame with a circular cross-section, the filament is wound at a constant α angle. However, if the filament is wound on a torus-shaped frame section, the wound $\tilde{\alpha }$ angle changes and is valid ${\tilde{\alpha }}_{int}<\alpha <{\tilde{\alpha }}_{ext}$. □

### 2.5. Determination of Torodial Helix Parameter ω

One of the parameters defining the expression of the toroidal helix in Relation (10) is a real ω value. If ω is a natural number, it indicates the number of turns of the toroidal helix on the whole torus. According to Relations (11) and (12), $\frac{2\pi R}{\omega }=2\pi r\cdot tg\;\alpha \;$ holds. From here it follows

(22) $$\omega =\frac{2\pi R}{2\pi r\cdot tg\alpha }=\frac{R}{r\cdot tg\alpha }.$$

The ω value determined by Relation (22) and used in the toroidal helix parametric Expression (10) ensures that the central axis o of the frame will pass through the winding head at the same speed when passing through both the straight and curved torus-shaped parts of the frame. In this case, the length ϑ on the o-axis at one turn of the straight frame part of the frame is equal to the length H on the o-axis at one turn of the toroidal helix on curved part of helix.

### 2.6. Optimal Number of Rovings Used during Winding

When winding the frame using rovings, it is desirable to ensure the following properties of the wound layer: the winding does not contain any gaps on the outer part of the torus, overlaps of adjacent rovings on the inner part of torus are minimized, and the approximate desired winding angle is maintained. The determination of the appropriate number of rovings when winding a curved torus-shaped frame section is the subject of this paragraph. 

First, attention is paid to determining the length of the arc on the circle $p_{1}$ at one turn of the toroidal helix (i.e., the length of the arc with the starting point $T_{0}$ and the ending point $T_{2}\;$on the circle $p_{1}$; see Figure 10b). Similarly, the length of the arc on the circle $p_{2}$ with starting point $T_{1}$ and ending point $T_{3}$ will be determined.

**Figure 11:**
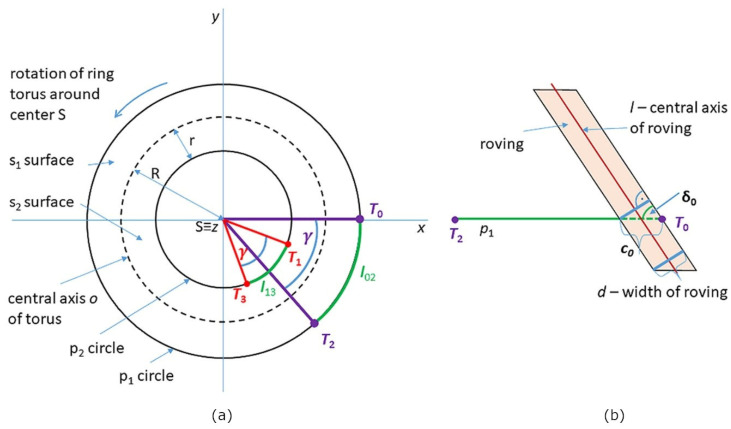
(**a**) Arc length part $l_{02}$ of circle $p_{1}$ and arc length part $l_{13}$ of circle $p_{2}$. (**b**) Laying a roving of width d at an angle ${\tilde{\alpha }}_{ext}\;$ (relation 18a/). Value $c_{0}$ indicates the length of the wound roving on connecting points $T_{0}$ and $T_{2}$.

From the parametric expression of the coordinates of points $T_{0}$, $T_{1}$, $T_{2}$ and $T_{3}$ in Relation (14) it is clear that the vectors $\vec{{ST}_{0}}$ and $\vec{{ST}_{2}}$ (see Figure 11a) are at an angle $\gamma =\frac{2\pi }{\omega }$ and analogously vectors $\vec{{ST}_{1}}$ and $\vec{{ST}_{3}}\;$are also at the same angle γ. At one turn of the toroidal helix, point $T_{0}\in p_{1}$ corresponds to point $T_{2}\in p_{1}$ and point $T_{1}\in p_{2}$ corresponds to point $T_{3}\in p_{2}$. The arc length $l_{02}\;$of circle $p_{1}$ with origin point $T_{0}$ and end point $T_{2}$ is given by relation (see [29], p. 11)

(23) $$l_{02}=\gamma \cdot \;(R+r).$$

Angle γ is given in Relation (23) in arc measure. Analogously the arc length $l_{13}\;$of circle $p_{2}$ with origin point $T_{1}$ and end point $T_{3}$ is given by the relation

(24) $$l_{13}=\gamma \cdot \;(R-r).$$

From Relations (23) and (24), it follows that the difference g of the arc lengths $l_{02}$ and $l_{13}$ is equal to $g=l_{02}-l_{13}=2\gamma r$. As the g-value increases, it becomes more difficult to ensure quality winding of the rovings on the curved part of the frame. 

Let d denote the width of the roving (see Figure 8b). The appropriate number of rovings when winding the curved part of the frame is determined by making one turn of the toroidal helix. Recall that $\delta_{0}$ is the angle that the tangent vector $w\left(0\right)$ of the toroidal helix makes with the tangent vector u(0) of the circle $p_{1}$ at the point $T_{0}$ (see Figure 10b, Relation (17)). It is valid (see Figure 11b) that $\sin\delta_{0}=d/c_{0},$ where $c_{0}$ denotes the width of the wound roving on the circle $p_{1}$. From here it follows

(25) $$c_{0}=d/\sin\delta_{0}\;.$$

Thus, the optimized number n of rovings used during torus winding is equal to

(26) $$n=\left\lceil \frac{l_{02}}{c_{0}}\right\rceil .$$

**Note 3.**

The ceiling $\left\lceil x\right\rceil$ of a real number, x, is defined as $\left\lceil x\right\rceil =min\left\{p\in Z;p\geq x\right\}$, where Z denotes a set of integers.

Further the total sum $\varepsilon_{02}$ of overlaps of adjacent rovings on the circle $p_{1}$ when winding a layer of rovings within one turn of the toroidal helix is equal to

(27) $$\varepsilon_{02}=n\cdot \;c_{0}-l_{02}.$$

The overlap ${\tilde{\varepsilon }}_{02}$ of two adjacent rovings on the circle $p_{1}$ is then equal to

(28) $${\tilde{\varepsilon }}_{02}=\frac{\varepsilon_{02}}{n}$$

Similarly, the angle $\delta_{1}$ at the point $T_{1}$ lying on the circle $p_{1}$ is defined. Parameter $c_{1}$ denotes the width of the wound roving on the circle $p_{1}$. Thus,

(29) $$c_{1}=d/\sin\delta_{1}\;\;$$

and the total sum $\varepsilon_{13}$ of overlaps of adjacent rovings on the circle $p_{2}$ within one turn of the toroidal helix is equal to

(30) $$\varepsilon_{13}=n\cdot c_{1}-l_{13}.$$

The overlap ${\tilde{\varepsilon }}_{13}$ of two adjacent rovings on the circle $p_{2}$ is then equal to

(31) $${\tilde{\varepsilon }}_{13}=\frac{\varepsilon_{13}}{n}\;.$$

Relations (26)–(30), (31) allow us to determine the optimized number of rovings when winding the torus. For a given roving width d, the minimum number of rovings used in the winding process can be determined. This prevents the formation of gaps between the rovings and at the same time ensures minimum overlaps between adjacent rovings on the outer circumference of the torus (circle $p_{1}$). At the same time, overlaps of adjacent rovings on the inner circumference of the torus (circle $p_{2}$) are minimized.

When using n rovings (n is defined by Relation (26)) of width d when winding the curved part of the torus-shaped frame, the *n* coils with wound rovings are distributed evenly around the circumference of the rotating ring of the winding head (see Figure 3). When winding the curved part of the frame, the relationships given in Section 2.6 apply. After the transition to the straight part of the frame, the rovings are wound at the desired α angle. The process of winding rovings onto a straight frame is discussed in detail in [26] and [25].

**Note 4.**

**Figure 12:**
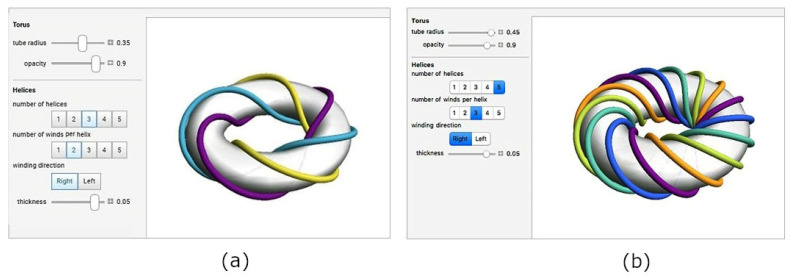
Examples of regular right-handed toroidal (**a**) 3-helix: major radius *R* = 10, minor radius *r* = 2.5, ω= 2 (number of winds per helix), (**b**) 5—helix: major radius *R* = 10, minor radius *r* = 4.5; ω = 3.

Relation (10) defines a toroidal helix wound on the torus. Consider hereafter only the central axes *l* of n rovings (see Figure 8b) wound on the torus. Following this, these axes form on the torus *regular toroidal n-helix* ([28]; see Figure 12).

## 3. Results and Discussion

This chapter focuses on the practical applications of derived relationships presented in the previous paragraphs. 

### 3.1. Determining the Difficulty of Torus Winding

As mentioned in the previous part of the article, winding a curved frame section with a circular cross-section is one of the most difficult parts of winding technology. Often the curved part of the frame is shaped in 2D and forms part of the torus (see Figure 5a). The three basic characteristics of the difficulty of performing a quality roving winding on a torus are applied in Table 1. The first column contains the values of major radius *R* and the second column minor radius *r* of the torus (see Figure 6a). The third column shows the values of aspect ratio a defined by Relation (3). The smaller the value of the parameter a is, the more suitable the conditions for winding (at a→ 0 the torus becomes a straight frame). The penultimate column contains the ratio of the surface area of the inner part $s_{2}$ and the outer part $s_{1}$ of the total torus surface (Figure 5a). The closer the ratio $s_{1}/s_{2}$ is to 1, the more suitable the torus is for winding. The last column shows the values of the ratios $o(p_{2})/o(p_{1})$. Here $o(p_{1})$ denotes the circumference of the circle $p_{1}$ on the outer circumference of the torus (Figure 6b) and $o(p_{2})$ the circumference of the circle $p_{2}$ on the inner circumference of the torus. Again, the closer the ratio $o(p_{2})/o(p_{1})$ is to 1, the better winding can be achieved.

**Figure 13:**
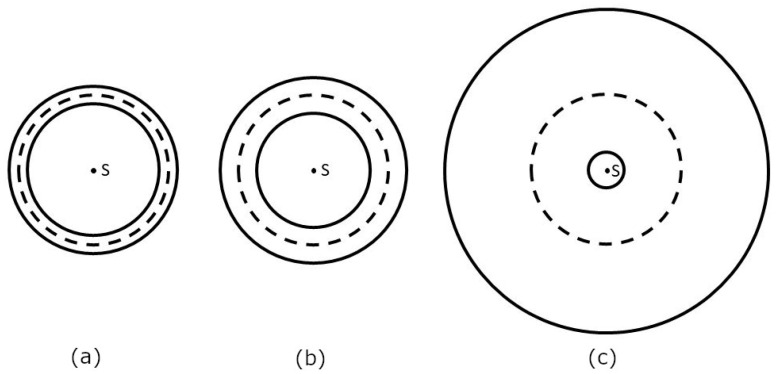
Floor plans of tori with parameters: $R=500\left[mm\right]$; (**a**) r=50 [mm], a=0.1; (**b**) r=100 [mm], a=0.2; (**c**) r=400 [mm], a=0.8.

Figure 13 shows three torus floor plans for the given pairs of R and r values from the fourth to sixth rows of Table 1. From Figure 13 and Table 1, it is clear that the best way to wind rovings onto the torus is in the a/ case and the worst way is in the c/ case.

If the winding difficulty characteristics are unfavorable for the specified torus, then it is appropriate to consider either another production technology or the use of a differently shaped frame for the manufacture of the composite. 

**Figure 14:**
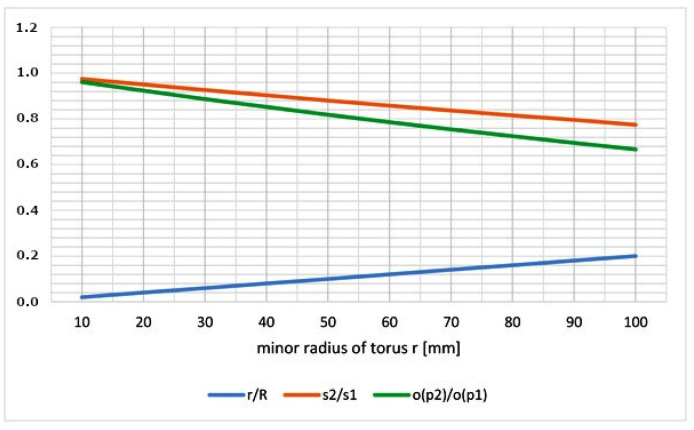
Graphical representation of a=r/R, $s_{2}/s_{1}$, and $o(p_{2})/o(p_{1})$ values for constant major radius R=500 [mm] and gradually increasing minor radius r.

Figure 14 shows the values of the characteristics a=r/R (Relation (3)), $s_{1}/s_{2}$ (Relation (6)) and $o(p_{2})/o(p_{1})$ (relation (8)). The best conditions for winding the roving on the torus occur in the case of a→0, $s_{2}/s_{1}\to 1$ and $o(p_{2})/o(p_{1})\to 1$.

It can be clearly seen from Figure 14 that as the value of r increases, the conditions for making a high-quality winding gradually deteriorate (aspect ratio of torus a=r/R gradually increases and values of $s_{2}/s_{1}$ and $o(p_{2})/o(p_{1})$ gradually decrease). It is possible to use any quantity of a=r/R, $s_{2}/s_{1}$, and $o(p_{2})/o(p_{1})$ as a measure of the difficulty of the winding, but we recommend the ratio $o(p_{2})/o(p_{1})$ as the most practically oriented measure.

### 3.2. Relations between Winding Parameters

The interrelationships of some parameters in winding the straight part of the frame and the curved part of the frame in the shape of a torus section are shown. An example of a frame composed of two straight parts and one curved part in the shape of a torus section is shown in Figure 5b. Table 2 gives examples of different parameter values for a frame with a circular cross-section, which includes a straight part and a curved part in the shape of a torus section. The first column contains the value of the major radius R and several different values of the minor radius r of the torus in the second column. Aspect ratio a is defined by Relation (3). The following two columns contain the different values of the α angle under which the roving winding is required and the corresponding values of the tangent function. The penultimate column contains the values of ω parameter defined by Relation (22); this parameter is significant in the toroidal helix parametric Expression (10). 

The last column of Table 2 contains values of toroidal pitch H calculated by the use of Relation (11).

Central axis o of the frame passes through the winding ring of the head at a constant speed. The required winding angle can be achieved by regulating the angular speed of the rotating ring of winding head when winding the straight part of the frame (angular speed is controlled by the robot’s external axis; for details see [25]). When winding a part of the torus-shaped frame, the winding angle changes during one turn in the range of values ${\tilde{\alpha }}_{int}$ to ${\tilde{\alpha }}_{ext}$ defined by Relations (20) and (21). Maintaining the same angular speed of the rotating ring of head when winding the bent part of the frame corresponds to the determination of the ω parameter using Relation (22). Parameter ω is applied in the parametric expression of the toroidal helix in Expression (10). The ω parameter defines the toroidal pitch H (specifies the length of repetition along the centre axis o) by Relation (11). 

### 3.3. Winding Angle of Rovings on the Torus

Based on the values of major radius R, minor radius r of the torus, and the desired winding angle  α on the frame, the winding angle ${\tilde{\alpha }}_{int}\;$of the roving on the inner circumference $p_{2}$ of the torus (see Figure 6b) and on the outer circumference $p_{1}$ of the torus ${\tilde{\alpha }}_{ext}$ can be determined. It always holds that ${\tilde{\alpha }}_{int}<\alpha <{\tilde{\alpha }}_{ext}$. Therefore, the winding angle ${\tilde{\alpha }}_{ext}$ on the outer circumference of the torus is larger than the winding angle ${\tilde{\alpha }}_{int}$ on the inner circumference of the torus. The angle of winding $\tilde{\alpha }\;$roving on the torus surface changes continuously from ${\tilde{\alpha }}_{int}\;$to ${\tilde{\alpha }}_{ext}$ and vice versa,$\;{\tilde{\alpha }}_{int}\leq \tilde{\alpha }\leq {\tilde{\alpha }}_{ext}$.

In Table 3, Relation (22) is used to calculate the parameter ω, Relations (18) and (21) are used to determine the angle ${\tilde{\alpha }}_{int}$, and Relations (17) and (20) are used to determine ${\tilde{\alpha }}_{ext}$.

Table 3 clearly shows that the deviations ${\tilde{\alpha }}_{int}$ and ${\tilde{\alpha }}_{ext}$ from the specified winding angle *α* increase with increasing aspect ratio *a*.

### 3.4. Determination of Optimal Number of Rovings

Based on the knowledge of the winding of the rovings on the frame from a geometrical point of view, the optimal number of rovings used in winding the new layer can be determined. Knowledge of the major radius R and minor radius r of the torus and the prescribed winding angle α is assumed. As shown in the previous Section 3.3, when the roving is wound onto the torus, the circumference $o(p_{1})$ (see Figure 6b and Figure 11a) is larger than the circumference $o(p_{2})$. At the same time, it was shown that on the outer circle $p_{1}$ the roving is wound at a larger angle ${\tilde{\alpha }}_{ext}$ than on the inner circle $p_{2}$ with angle ${\tilde{\alpha }}_{int}$.

For the optimum number n of rovings to be used for roving width d, it is desired to create a winding without gaps and with zero or minimal overlap of two adjacent rovings on the outer circle $p_{1}$. At the same time, the size of the overlap of two adjacent rovings can be determined on the inner circle $p_{2}.$ This overlap is always larger than on $p_{1}$. 

Table 4 shows the calculated values n of the optimal number of rovings used for a given width d and values of R, r, and winding angle α. From the knowledge of values R, r, α and Relation (22), the parameter ω can be determined. At the same time, the overlaps of two adjacent rovings on the outer circumference $p_{1}$ and the inner circumference $p_{2}$ are determined. By successively using Relations (17), (23), (25)–(28), the overlap ${\tilde{\varepsilon }}_{02}.$ of two adjacent rovings on the outer circumference $p_{1}$ of the torus can be determined. Similarly, by successively using Relations (18), (24), (29)–(31), the overlap ${\tilde{\varepsilon }}_{13}$ on the inner circumference $p_{2}$ of torus can also be determined.

**Note 5.**

The carbon rovings are from Toho Tenax, a widespread manufacturer of winding rovings. Carbon roving 24 K consists of twenty-four-thousand carbon filaments about a diameter 7 [μm], creating a rectangular cross-section with a width of 9 [mm]. Carbon rovings marked 12 K and 6 K have a width of 12 K = 5 [mm] and 6 K = 2 [mm].

**Figure 15:**
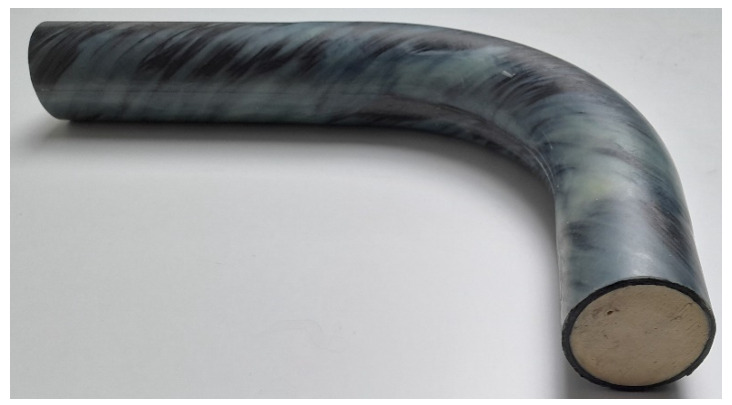
Example of the curved part of polymer composite frame with the following parameters: major radius *R* = 102.5 [mm], minor radius *r* = 17.5 [mm], width of roving *d* = 5 [mm]. The non-load-bearing polyurethane frame is visible in the vertical section (light colour of the cross-section).

The curved part of a polymer-composite frame after the simultaneous successive winding of three layers of carbon rovings on a non-load-bearing frame under specified winding angles *α* equal to 45°, −45°, and 45° is shown in Figure 15. The curved part of the frame forms one-quarter of the torus. Subsequently, the wound frame is thermally treated.

### 3.5. Recommended Procedure before Starting Winding

Before starting the actual winding procedure on a frame with a curved section in the shape of a torus part, it is advisable to carry out the following preparatory steps.

1. Determine the suitability of winding the rovings on the non-load-bearing frame (Relations (3), (7), and (8) can be used, see Table 1). If the winding conditions are unfavorable, consider whether, for example, to use a differently shaped frame or to choose a different composite manufacturing technology.
2. Calculate parameter ω using Relation (22). Based on the knowledge of this parameter, an estimate of the number of roving revolutions on the whole toroidal helix can be obtained.
3. Determine the maximum winding angle of the roving ${\tilde{\alpha }}_{ext}$ on the torus at the outer circumference $p_{1}$ (see Figure 6b) using Relations (17) and (20). At the same time, determine the minimum winding angle ${\tilde{\alpha }}_{int}$ on the inner circumference of $p_{2}$ by applying Relations (18) and (21). For the required winding angle α for a given layer, the following relation holds: ${\tilde{\alpha }}_{int}<\alpha <{\tilde{\alpha }}_{ext}$. During the winding procedure, the winding angle $\tilde{\alpha }\;$on the torus changes continuously and ${\tilde{\alpha }}_{int}\leq \tilde{\alpha }\leq {\tilde{\alpha }}_{ext}$. Due to the continuously changing winding angle $\tilde{\alpha }$, it is useful to determine whether the changing winding angle satisfies the winding requirements with respect to the planned loading of the polymer composite frame using a suitable modelling software tool (e.g., ABAQUS, ANSYS).
4. Determine the optimized number of rovings n for the winding of the layers at their specified width d. To the selected value of n, calculate the overlap ${\tilde{\varepsilon }}_{02}$ of two adjacent rovings on the outer circumference $p_{1}$ and the overlap ${\tilde{\varepsilon }}_{13}$ on the inner circumference $p_{2}$. Following this, select the winding of the roving with the most suitable width d provided by the supplier of rovings.

Based on the above procedure, it is possible to define the optimized the winding procedure.

## 4. Conclusions

The article focuses on the problem of winding rovings on a non-load-bearing frame with a circular cross-section, and the problem of winding the curved part of the frame is solved. In particular, attention is paid to the case where the curved section of the frame forms part of the torus. The problem is solved from a geometric point of view. Based on the geometric parameters of the torus and the relations derived in the paper, the level of difficulty of the roving layer winding, including the real feasibility of homogeneous winding, can be determined. Based on the given torus and using the relations derived in Chapter 2, the parametric expression of the wound toroidal helix can be determined. As a result, the behavior of the roving when winding on a torus can be described analytically. The winding angle changes continuously within one turn of the roving.

In Section 2.2. we provide three alternative quantities, specifically a=r/R, $s_{2}/s_{1}$, and $o(p_{2})/o(p_{1})$, that describe the level of difficulty of winding on given toroidal part of the frame. However, we recommend the quantity $o(p_{2})/o(p_{1})$ as the most practically oriented measure of the winding difficulty.

When winding the roving layer, it is necessary to avoid gaps in the winding on the outer circumference of the torus and at the same time it is necessary to minimize overlaps of adjacent rovings on the inner circumference of the torus. Using the relations from the previous section, the optimal number of rovings used to wind the layer onto the torus can be determined for a given roving width. At the same time, the overlap size of two adjacent wound rovings can be determined. 

A greater number of rovings and their shorter length are required when the frame is wound at a greater angle (for a curved section of the frame in the shape of a torus section, a smaller ω parameter corresponds to a greater winding angle). When winding at a smaller winding angle, fewer rovings of greater length are required. The total amount of material required is practically the same, unless we consider the issue of overlapping adjacent rovings. Of course, different frame loads (tension, torsion, etc.) correspond to different suitable winding angles. Practical tests show that it is not advisable to wind the roving on the torus at an angle greater than 45°, as this usually causes the roving to “slide” on the surface of the frame and degrade the entire winding.

Meeting the necessary geometric conditions of winding is a prerequisite for a quality winding of the roving layer. As the frame passes through the winding head, three layers of roving are wound simultaneously at different angles (the winding head contains three rotating rings with coils of wound roving). If more layers of windings are required on the frame, the frame can be passed through the winding head repeatedly.

The problems of winding straight frames with circular cross-sections (especially the smooth transition to another winding angle, the distance of winding roving from the rotating ring of the winding head, and the determination of the optimal number of rovings when winding a layer of rovings) are analyzed mainly in previous published works [25,26]. These articles, together with this paper, comprehensively describe the problem of winding composite frames using rovings. The fulfillment of the required geometrical conditions of winding is a prerequisite for ensuring the production of high-quality polymer composite frames. A detailed procedure for calculating the optimal trajectory of the industrial robot during the winding process even for curved frames is described in [23,33].

## Figures and Tables

**Table 1:** Characteristics indicating the level of difficulty of performing a quality roving winding.

Major Radius (*R*) [mm]	Minor Radius (*r*) [mm]	Aspect Ratio (*a*)	Ratio $\frac{s_{2}}{s_{1}}$	Ratio $\frac{o(p_{2})}{o(p_{1})}$
1000	20	0.02	0.9748	0.9607
	500	0.5	0.5171	0.3333
	800	0.8	0.2407	0.1111
500	50	0.1	0.8802	0.8181
	100	0.2	0.7741	0.6666
	400	0.8	0.3251	0.1111
100	20	0.2	0.7741	0.6666
	50	0.5	0.5171	0.3333
	90	0.9	0.2715	0.0526
50	10	0.2	0.7741	0.6666
	20	0.4	0.5941	0.4285
	30	0.6	0.4472	0.2500

**Table 2:** Interrelation of parameters when winding the straight part of the frame and the curved part of the frame in the shape of the torus part.

Major Radius (*R*) [mm]	Minor Radius (*r*) [mm]	Aspect Ratio (a)	Winding Angle (α) [°] [rad]		tg α	Parameter ω	Toroidal Pitch (H) [mm]
500	25	0.05	5	0.0815	0.0874	228.8329	13.7287
			30	0.5235	0.5773	34.6440	90.68215
			45	0.7853	1.0000	20.0000	157.0796
	50	0.1	5	0.0815	0.0874	114.3118	27.4826
			30	0.5235	0.5773	17.32051	181.3799
			45	0.7853	1.0000	10.0000	314.1592
	100	0.2	5	0.0815	0.0874	61.3496	51.2080
			30	0.5235	0.5773	8.6610	362.7286
			45	0.7853	1.0000	5.0000	628.3185
	450	0.9	5	0.0815	0.0874	12.7128	247.1204
			30	0.5235	0.5773	2.1224	1480.2076
			45	0.7853	1.0000	1.1111	2827.4616

**Table 3:** Determination of the winding angle ${\tilde{\alpha }}_{int}$ on the inner circumference of the torus (circle $p_{2}$ ) and the winding angle ${\tilde{\alpha }}_{ext}$ on the outer circumference of the torus (circle $p_{1}$ ) depending on the major radius R of the torus, the minor radius r of the torus and the desired winding angle α.

Major Radius (*R*) [mm]	Minor Radius (*r*) [mm]	Aspect Ratio (*a*)	Winding Angle (α) [°] [rad]		tg α	Parameter ω	Angle ${\tilde{\alpha }}_{int}$ [°]	Angle ${\tilde{\alpha }}_{ext}$ [°]
100	20	0.2	5	0.0815	0.0874	57.2082	3.9968	5.9872
	50	0.5	30	0.5235	0.5773	3.4644	16.1007	40.8909
	90	0.9	45	0.7853	1.0000	1.4148	4.4904	56.1712
50	10	0.2	5	0.0815	0.0874	61.3496	3.73040	5.5857
	20	0.4	30	0.5235	0.5773	4.7755	17.4376	36.2379
	30	0.6	45	0.7853	1.0000	1.6666	21.8021	57.9956

**Table 4:** Optimized number of rovings n used in winding and the size of overlaps ${\tilde{\varepsilon }}_{02}$ on the outer and ${\tilde{\varepsilon }}_{13}\;$ on the inner circumference of the torus for given values of R, r, d and α.

Outer Radius (*R*) [mm]	Inner Radius (*r*) [mm]	Param. *a*	Angle Winding (α) [°]	Param. ω	Roving Width (*d*) [mm]	Optimized Number of Rovings (*n*)	Outer Overlap ( ${\tilde{\varepsilon }}_{02}$) [mm]	Inner Overlap (${\tilde{\varepsilon }}_{13}$) [mm]
100	20	0.2	10	28.3607	9	3	0.3378	3.1811
	25	0.25	30	6.9282		11	0.7944	3.6238
	30	0.3	45	3.3333		17	0.3467	3.2236
200	10	0.05	10	113.4429	5	3	1.2083	1.5621
			30	34.6410		7	0.4055	0.7795
			45	20.0000		10	0.6526	0.9274

## Data Availability

Not applicable.
